# Low secondary attack rate after prolonged exposure to sputum smear positive miliary tuberculosis in a neonatal unit

**DOI:** 10.1186/s13756-022-01179-8

**Published:** 2022-12-05

**Authors:** Roxana Pop, Marisa B. Kaelin, Stefan P. Kuster, Hugo Sax, Silvana K. Rampini, Reinhard Zbinden, Christa Relly, Bea Zacek, Dirk Bassler, Jehudith R. Fontijn, Christoph Berger

**Affiliations:** 1 Department of Infectious Diseases and Hospital Hygiene, University Hospital Zurich, University of Zurich, Raemistrasse 100, 8091 Zurich, Switzerland; 2 Department of Infectious Diseases, Bern University Hospital, University of Bern, Bern, Switzerland; 3 Department of Internal Medicine, University Hospital Zurich, University of Zurich, Zurich, Switzerland; 4 Institute of Medical Microbiology, University of Zurich, Zurich, Switzerland; 5 University Children’s Hospital Zurich, Division of Infectious Diseases and Hospital Epidemiology, University of Zurich, Zurich, Switzerland; 6 TB Centre of the Lung Association of Canton Zurich (Verein Lunge Zürich), Zurich, Switzerland; 7 Department of Neonatology, University Hospital Zurich, University of Zurich, Zurich, Switzerland

**Keywords:** Tuberculosis, Neonatal intensive care unit, Neonate, Infant, outbreak investigation, Interferon gamma release assay, T-Spot, Quantiferon, TST

## Abstract

**Background:**

Several neonatal intensive care units (NICU) have reported exposure to sputum smear positive tuberculosis (TB). NICE guidelines give support regarding investigation and treatment intervention, but not for contact definitions. Data regarding the reliability of any interferon gamma release assay (IGRA) in infants as a screening test for TB infection is scarce. We report an investigation and management strategy and evaluated the viability of IGRA (T-Spot) in infants and its concordance to the tuberculin skin test (TST).

**Methods:**

We performed an outbreak investigation of incident TB infection in a NICU after prolonged exposure to sputum smear positive miliary TB by an infant’s mother. We defined individual contact definitions and interventions and assessed secondary attack rates. In addition, we evaluated the technical performance of T-Spot in infants and compared the results with the TST at baseline investigation.

**Results:**

Overall, 72 of 90 (80%) exposed infants were investigated at baseline, in 51 (56.7%) of 54 (60%) infants, follow-up TST at the age of 6 months was performed. No infant in our cohort showed a positive TST or T-Spot at baseline. All blood samples from infants except one responded to phytohemagglutinin (PHA), which was used as a positive control of the T-Spot, demonstrating that cells are viable and react upon stimulation. 149 of 160 (93.1%) exposed health care workers (HCW) were investigated. 1 HCW was tested positive, having no other reason than this exposure for latent TB infection. 5 of 92 (5.5%) exposed primary contacts were tested positive, all coming from countries with high TB incidences. In total, 1 of 342 exposed contacts was newly diagnosed with latent TB infection. The secondary attack rate in this study including pediatric and adult contacts was 0.29%.

**Conclusion:**

This investigation highlighted the low transmission rate of sputum smear positive miliary TB in a particularly highly susceptible population as infants. Our expert definitions and interventions proved to be helpful in terms of the feasibility of a thorough outbreak investigation. Furthermore, we demonstrated concordance of T-Spot and TST. Based on our findings, we assume that T-Spot could be considered a reliable investigation tool to rule out TB infection in infants.

## Background

### Introduction

Tuberculosis (TB) exposure is a recognized risk in health care settings. Data regarding transmission within neonatal intensive care units (NICUs) are scarce. Newborns are particularly susceptible to TB infection and disease [1], especially the risk of disseminated disease, with potentially fatal consequences, is increased. Therefore outbreak investigations require special attention [2]. However, standardized protocols regarding contact definition are lacking. The NICE Guidelines offer direction regarding investigation management and the required interventions after exposure [3]. They recommend tuberculin skin test (TST) as well as any interferon-gamma release assay (IGRA) as a diagnostic tool in their algorithm. Generally, T-Spot is considered to be more sensitive than Quantiferon in immunosuppressed population [4]. As cell-mediated immune response in children, especially among those younger than 5 years of age, is still developing, and because the T-Spot can be performed with a smaller volume of blood, the T-Spot is the preferred diagnostic tool [5]. Nevertheless, few data exist regarding the performance of any IGRA as an investigation tool for TB infection in infants and children below 5 years of age and the results are more often reported as indeterminate compared to adults [6].

### Objectives

We report a prolonged exposure to a sputum smear positive miliary TB and a potential strategy of investigation management after exposures in a NICU. In an outbreak investigation, we assessed secondary attack rates of infants, health care workers (HCW) and other primary contacts. In addition, we evaluated the technical performance of IGRA (T-Spot) and the concordance of its results with TST at baseline investigation in this large cohort of infants.

### Case description of the index patient

A 24-year-old HIV-negative pregnant woman, originally from Guinea, with a history of cough and vaginal bleeding for over 2 weeks, was admitted to the obstetric ward. Because of suspected amniotic infection syndrome, a Caesarean section had to be performed with 24 3/7 weeks of pregnancy and the preterm neonate was admitted to the NICU for further treatment and care.

After delivery, the mother suffered from persistent abdominal pain and cough. Two months later, an emergency laparoscopy had to be performed because of the suspicion of a tubo-ovarian abscess. Intraoperative situs showed amber colored fluid collection and adhesions. Swabs from the fluid for Ziehl–Neelsen staining, polymerase chain reaction (PCR) and culture were negative for *Mycobacterium tuberculosis* (MTB) complex. Tissue biopsy revealed a non-necrotizing granulomatous inflammation. Finally, a CT-scan of the abdomen and thorax, performed after 3 months of further illness, disclosed the suspected diagnosis of a miliary tuberculosis. Ziehl–Neelsen-staining of the sputum showed acid-fast bacilli, PCR for *M. tuberculosis* complex DNA was positive and culture showed growth of *Mycobacterium africanum*. At the time of diagnosis, her newborn was already discharged from the NICU.

Retrospectively, a PCR from placental tissue was negative, but the PCR of the abdominal tissue biopsy taken during the laparoscopy was positive for *M. tuberculosis* complex DNA. In summary, the miliary TB was diagnosed with a delay of 5 months. Critical anamnestic facts, such as the patient's country of origin, were not considered, and the initial diagnostic workup was inappropriate.

The mother’s visits were not uniformly documented regarding time and duration. However, the documentation confirmed that she visited her preterm born infant regularly, mostly daily. During her visits, she wore a surgical face mask only when a of cough was obviously present.

## Method

### Study design

We performed an outbreak investigation in a NICU of a tertiary care university hospital, assessing secondary attack rates as well as concordance of T-Spot and TST in infants after prolonged TB exposure. The neonatal department comprises an intensive care and an intermediate care unit with a maximal capacity of 36 beds. Intensive care patients, as well as infants in transition to intermediate care, are cared for in two not fully separated large units with a total capacity of 20 beds (closed incubators as well as open cots). The outbreak investigation took place before the COVID-19 pandemic. Accordingly, face masks were not routinely used.

As there is neither a consensus case definition in the scientific literature nor a definition for infants considered as contacts, the ad-hoc outbreak management team, consisting of experts in infection prevention and control, neonatology and pediatric infectious diseases, established definitions for case and contacts and steered the diagnostic and therapeutic interventions (see Table 1).

**Table 1:** Definitions of contacts and interventions used

Contacts	Contact definition	Investigation period	Diagnostic and therapeutic interventions
Infants	Every neonate admitted to NICU for > 24 h during the same period as the index patient’s infant	Entire period during admission of the index patient’s infant, regardless the time period to the diagnosis	TST and T-Spot at baseline, TST after 6 months of exposure No prophylactic treatment, as exposure was at least 6 weeks ago at time of investigation
Relatives of contact infants	Every relative of a neonate admitted for > 4 days at the NICU during the same period as the index patient’s infant	Until 3 months before diagnosis and initiation of therapy of the index patient	TST or Quantiferon 8 weeks after last exposure, CXR in case of positive result Isoniazid or rifampicin in case of newly diagnosed latent TB
Family members	Every family member	Na	Adults: TST or Quantiferon 8 weeks after last exposure, CXR in case of positive result Isoniazid or rifampicin in case of newly diagnosed latent TB Children: TST(& IGRA in the newborn), chest X-ray and gastric lavage at baseline and TST 8 weeks after last exposure; Isoniazid up to second negative TST
Other patients	Every patient sharing the same room as the index patient for more than 8 h during the index patients instays	Until 3 months before diagnosis and initiation of therapy of the index patient	TST or Quantiferon 8 weeks after last exposure, CXR in case of positive result Isoniazid or rifampicin in case of newly diagnosed latent TB
Health care workers	Close nursing contact to the index patient or cumulative contact for more than 8 h to the index patient’s infant or herself	Entire period during admission of the index patient’s infant, and during her own instays, regardless the time period to the diagnosis	TST or Quantiferon 8 weeks after last exposure, CXR in case of positive result Isoniazid or rifampicin in case of newly diagnosed latent TB

### Exposure investigation

#### Definitions

##### Index patient

As the mother was Ziehl–Neelsen smear positive (+++), *M. tuberculosis* complex DNA polymerase chain reaction (PCR) positive and culture positive for *M. africanum,* we defined her as the index patient. Regarding the exact course of the mother’s disease, we assumed that she was contagious from the onset of respiratory symptoms, even before giving birth. Her infant was not considered as an index case, but as severely exposed.

##### Contacts

According to the Swiss national TB guidelines and European Consensus Guidelines, every immunocompetent person being in contact with an sputum smear positive TB case for more than 8 h is considered exposed [7, 8]. Immunosuppressed persons and children aged 12 or younger generally are at high risk for acquiring tuberculosis [7–10]. Therefore, at the study hospital, immunosuppressed persons and children aged 12 or younger are considered exposed when having shared the same room with the index patient, regardless of the duration of exposure.

In addition, we followed the European consensus board, who decided that a relevant risk of exposure should be evaluated for persons who have been in contact with the index patient during the period of 3 months before diagnosis and initiation of treatment of the index patient [7]. In case of a confirmed incident TB infection, the investigation period would be expanded back to the onset of symptoms of the index patient. But, as exposed infants are at increased risk of TB infection or disease, and infections of HCWs could have a huge impact on infection control and hospital acquired infections, we did not apply this temporal criterion to infants and HCWs. We decided to investigate all exposed infants and HCWs from the onset of symptoms of the index patient over a period of 5 months (see Table 1 “investigation period”).

- Infants.

Although the mother’s visits to the NICU were not uniformly documented regarding time and duration, she visited her infant mostly daily for 1–2 h over a period of 3 months. Therefore, every child hospitalized simultaneously in the NICU with the index patient’s child for more than 24 h was regarded as a contact.

(b) Health care workers.

HCW with close nursing contact to the index patient as well as HCW working in the patient’s room or with her infant for more than 8 h in total were considered as contacts.

(c) Primary contacts other than infants or healthcare workers:
  Family members: The husband, the newborn and the other children of the index patient, aged 3 and 5 years, as well as the social worker accompanying the family were considered as contacts.
  Relatives of contact infants: Taking into account the index patient’s visits, every visiting relative of an infant hospitalized for more than 4 days during the same period as the child of the index patient, was assumed to be a contact.
  Other patients: Every patient sharing a room with the index patient during her two inpatient-stays (Caesarean section and laparoscopy) for more than 8 h was considered a contact.

### Diagnostic and therapeutic interventions

#### Infants

##### Infants hospitalized in the NICU

The miliary TB of the index patient was diagnosed about 6 weeks after discharge of her newborn from the NICU. Since most of the infants were released from the NICU at the time the index patient was diagnosed, we informed parents of the contact infants, as well as their pediatricians, by mail and asked the parents to participate in exposure investigation of their infant. In case of exposure, NICE Guidelines recommend an immediate start of isoniazid (INH) prophylaxis and its reevaluation after 6 weeks along the results of performed TST or IGRA. As in our case, the time of exposure was at least 6 weeks prior, we did not start INH prophylaxis. T-Spot and TST were performed at baseline in every contact infant. For the purpose of reassurance, we conducted a second TST in all infants at the chronological age of 6 months (except the infant reached this age already at the time of the first investigation). We did not perform a routine second T-Spot as we wanted to avoid inconvenience of phlebotomy in small children. In case of a positive TST we would have performed a T-Spot for comparison. Further diagnostics were planned in case of clinical signs consistent with an active TB disease or a positive TST or T-Spot. Since the diagnostic value of an X-ray is limited, we did not perform chest X-rays.

##### Family member infants

For the index patient’s newborn child and for the two siblings, we assumed an intensive and permanent exposure until the time of diagnosis of the index patient. Therefore, the time criterion (of 6 weeks) could not be applied. TST and T-Spot was performed in all three children. To exclude active tuberculosis safely, chest X-ray and gastric lavage was performed in all three children. According to Swiss guidelines, all three siblings received isoniazid prophylaxis immediately after the mother was diagnosed and active TB was ruled out in the newborn. After 8 weeks, we repeated TST in all three children and stopped the INH prophylaxis due to the persistent negative results.

#### Primary contacts other than infants

We followed the Swiss national TB guidelines and performed TST or IGRA (Quantiferon), depending on previously existing result, 8 weeks after the last exposure. A chest X-ray was performed in case of a positive result. Isoniazid or rifampicin prophylaxis was established in case of a newly diagnosed latent TB infection (LTBI).

### Microbiological assays

T-Spot. TB (T-Spot, Oxford Immunotec, Abingdon, Oxfordshire, UK) and Quantiferon TB Gold Plus enzyme-linked immunosorbent assay (ELISA) (QFT®Plus, Qiagen GmbH, Hilden, Germany) were performed according to the prescriptions of the manufacturer. TST was performed according standard procedures [7].

#### T-spot and its viability

T-Spot is a variant of the Enzyme-linked immune Spot (ELISpot) technique that is designed for the detection of effector T-cells in heparinized patient blood stimulated by ESAT-6 (Panel A) and CFP-10 (Panel B). The test enumerates individual ESAT-6 and CFP-10 specific cells by measuring secreted interferon-γ (IFN-γ) around the effector T-cells by an ELISA resulting in a spot. A control tube (NIL) without antigens is performed to detect nonspecific cell activation, i.e., secretion of IFN-γ around the cells without any antigen stimulation. A positive control containing PHA confirms viability and functionality of the T-cells and must reach > 20 spots. The test is interpreted as positive or as negative if the number of spots of Panel A (ESAT-6) and/or Panel B (CFP-10) minus the number of the spots of the NIL control reveals ≥ 6 (positive) or ≤ 5 spots (negative), respectively.

The T-Spot results were compared to TST results at baseline (at least 6 weeks after last exposure).

We performed descriptive statistics to evaluate the viability of T-Spot in infants.

#### Quantiferon TB gold plus ELISA

QFT®Plus is an in vitro diagnostic test using peptide cocktails to stimulate cells in heparinized blood. Detection of secreted IFN-γ by ELISA is used to identify in vitro responses to those peptide antigens that are associated with *M. tuberculosis* complex infection. QFT ®Plus has two distinct tubes: TB-Antigen Tube 1 (TB1) with *M. tuberculosis* complex antigens ESAT-6 and CFP-10 that are stimulating mainly CD4 helper T-cells and TB-Antigen Tube 2 (TB2) with an additional set of peptides that stimulate cytotoxic CD8 cells. An additional tube (MITOGEN) with (PHA) stimulating T-cells unspecifically to produce IFN-γ is added as a positive control. Results > 0.5 IU/ml are required as a positive control. An additional NIL tube with heparinized blood without any antigens is added as negative control. After an incubation of 16 h the 4 tubes are centrifuged (15,2000*g*) and the supernatants are tested with the QFT®Plus ELISA. The results of the 4 tubes are compared with a standard curve of IFN-γ (IU/mL). Values of TB1-NIL or/and TB2-NIL > 0.35 are considered positive according to the manufacturer. According to in-house standards, we considered results of > 0.35 IU/mL but < 1 IU/mL as inconclusive and results ≥ 1 IU/ml as positive.

## Results

### Exposure investigation

#### Infants

Overall, 90 infants were exposed according to the contact definition used. The median gestational age in weeks was 32 6/7 (range 23 6/7–41 5/7), and the median weight at birth was 1950 gr. At the time of the first test, all infants had a postmenstrual age of at least 37 weeks. We conducted baseline TST and T-Spot in 72 of 90 (80%) exposed infants. Five (5.6%) infants died on the NICU of other causes than TB and six (6.6%) were lost to follow. Four (4.5%) infants were still hospitalized at the NICU so exposure investigation was done during hospitalization and seven (7.8%) infants were investigated elsewhere (TST only, no report of positive tests). Baseline TST and T-Spot were negative in all cases, except one, in which T-Spot was inconclusive.

17 (18.9%) infants had a chronological age of 6 months at the time of the first investigation. In these children, no follow-up investigation took place. In 51 (56.7%) of the 54 (60%) remaining infants, we performed a follow-up TST at the age of 6 months. Again, all infants were tested negative. Three (3.3%) infants were lost of follow-up before the second investigation.

#### Health care workers

Out of the 160 exposed HCW, 139 (86.9%) had a negative Quantiferon. 11 (6.9%) were lost to follow up. 10 (6.3%) HCW were diagnosed with latent TB, 5 of them were however already tested positive before. Out of the five newly diagnosed HCW for latent TB, two originate from countries with a high TB incidence, in two HCW’s Quantiferon were repeatedly inconclusive and one HCW showed a positive Quantiferon result. A normal chest X-ray and the lack of symptoms excluded an active TB disease. The HCW received a prophylactic therapy for latent TB with rifampicin for the duration of 4 months.

#### Primary contacts other than infants and health care workers

1. Family members: of the four family members tested for latent TB, only the husband of the index patient was tested positive. As the index patient, the husband originates from Guinea, which is considered a country with high TB high incidence. The time and duration of the latent infection of the husband cannot be specified.
2. Relatives of exposed infants: 86 relatives of infants were exposed, 80 (93%) were tested, 6 (7%) were lost to follow up. Four (4.7%) relatives were diagnosed with latent TB, all coming from TB high burden countries. The precise moment of TB infection in these cases is therefore unclear.
3. Other patients: two other exposed patients received TST or Quantiferon, none of them showed a positive result.

**Fig. 1:**
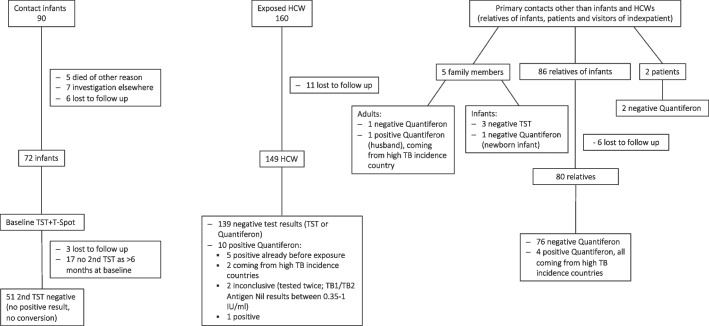
Summary of results of outbreak investigation

The exposure investigation is summarized in Fig. 1.

### Secondary attack rate

In total, one of 342 exposed contacts was newly diagnosed with latent TB infection. This corresponds to a secondary attack rate of 0.29%. Counting only the tested contacts, 1 of 304 was newly diagnosed with latent TB infection, corresponding to a secondary attack rate of 0.32%.

### Viability of T-spot in contact infants

In our baseline investigation, all infants showed a negative TST and a concordant negative T-Spot. All infants except one responded to PHA (positive controls revealed > 20 spots).

## Discussion

This report describes a TB exposure and outbreak investigation of a sputum smear positive miliary TB on a NICU with low secondary attack rate. To our knowledge, no report with an adult as index patient suffering from miliary TB in this setting has been published. In addition, this report shows good technical performance of the T-Spot in infants with concordant negative results of T-Spot and TST at baseline investigation in all tested infants in our cohort.

### Index patient and contact definitions

In the past years, several NICUs have experienced and reported exposure to sputum smear positive tuberculosis [2, 11–23]. In most reports, the index patient is a neonate suffering from congenital TB [2, 11–13, 20–23] although exposures through HCW are also described [12–14, 22, 23]. NICE Guidelines give support regarding investigation and treatment intervention, not though for contact definitions [3], leading to substantial differences in most reports regarding clinical presentation of the index patients and management protocols. Decisions regarding contact definitions and management protocols rely on expert opinions, as shown along a review of the literature we performed which is summarized in Table 2 [2, 11–23].

**Table 2:** Summary of reviewed literature

References	Year	Country	Index patient		Investigation		Intervention	Results infants		Results other primary contacts			Results HCW
			1	> 1	Infants	Adults		Pos. TST 1°	Pos. TST 2°	Pos. TST 2°	Conversion	Pos. TST 2°	Conversion
Lee et al. [22]	1997	USA (NYC)	infant with congenital TB; mother described as healthy		TST, gastric aspirate, CXR if already discharged	TST, CXR if TST positive	Isoniazid, Pyridoxine for 6 months	0/14	0/10	4/27	unknown	2/260	2/260
Grisaru-S. et al. [20]	2014	Israel	infant with congenital TB; mother with pulmonary TB		TST, CXR if TST positive	TST, CXR if TST positive	INR if < 3 months old or > 3 months and immunodeficient	0/97	1/75	32/156	11/80	48/115	3/41
Crockett et al. [11]	2004	Canada		2 infants with congenital TB *	TST, CXR, CT if abnormal CXR, gastric aspirate	TST, CXR	INH until TST negative, evaluation after 1 and 3 months	na	na	3/3	2/3	na	3/328
Isaacs et al. [19]	2006	Australia	mother with pulmonary TB		TST, CXR, gastric aspirate if abnormal CXR	TST, CXR	INH for 6 months, treatment of TB disease in high suspicion	0/13	na	2/20	2/20	0/40	0/40
Laartz et al. [23]	2002	USA (Florida)	Infant with congenital TB; mother with endometrial biopsy culture positive		TST, CXR if TST positive	na	INH for 3 to 6 months	0/36	0/30	1/106	1/106	0/120	0/120
Winters et al. [2]	2010	USA (NYC)	Infant congenital TB; mother sputum negative		TST, CXR	TST, CXR if TST positive	na	na	0/13	na	0/10	2/64	2/64
Ahn et al. [15]	2015	Korea	nurse, pulmonary TB		TST, CXR	TST, CXR	INH for 3 months	2/108	2/106	na	na	33/59	6/59
Fisher et al. [14]	2014	Australia	medical officer with smear positive pulmonary TB		TST (month 3 of age), CXR	TST, CXR	INH until negative TST	0/125	0/89	51/152	3/152	9/120	1/120
Nania et al. [16]	2007	USA (Nashville)	respiratory therapist		None	TST	none	na	na	0.5	0/5	0/223	0/223
Perry et al. [17]	2012	France (Paris)	caregiver, pulmonary TB (culture positive)		TST (month 0 and 3), CXR	na	INH and Rifampicin for 3 months	4/172	1/150	na	na	na	na
Mouchet et al. [12]	2004	Belgium (Brussels)	infant with congenital TB; mother: chest X-Ray negative		TST, gastric aspriate in some children, CXR	HCW: TST or CXR family members: CXR	INH to high risk infants	0/85	na	na	na	na	6/32
Steiner et al. [18]	1975	USA (NYC)	nurse aid, cavitary disease, sputum/culture negative, asymptomatic		TST, CXR if TST positive	TST, CXR in case of positive TST	none	2/1647; 2 with active disease	na	18/18	na	na	Na
Saitoh et al. [13]	2001	Japan	infant with congenital TB; mother with menstrual discharge positive		TST, CXR	TST, CXR	INH for 3 months only to the children at NICU	0/99	0/99	na	na	3/144	3/144
Lee et al. [21]	2004	USA (NYC)	infant with congenital TB		TST, CXR	TST, CXR in case of positive TST	INR for 3 months	0/11	1/15	na	na	4/211	4/211

^*^Child to child transmission via contaminated resp. equipment

In the here reported investigation, the mother was considered as index patient. The infant of the index patient had not shown symptoms consistent with a congenital TB, the placental PCR was negative for MTB complex, the result of the infant’s gastric lavage and the TST up to 6 months of age were all negative and therefore the diagnosis of a congenital and postnatal TB infection was unlikely.

Given the large number of infants in the NICU, the delayed diagnosis of the index patient’s disease, and the unexact documentation of the mother’s daily presence, we decided that every neonate, hospitalized more than 24 h, was exposed. The TB transmission risk for infants is minimal, not negligible though, and the potential harm of TB disease life threating [24]. This justified our, complex and time intensive outbreak investigation. In terms of feasibility and accurateness, our expert definitions proved to be useful in this thorough outbreak investigation.

### Therapeutic interventions

A considerable issue is the lack of evidence regarding the indication for the administration of isoniazid as a chemoprophylaxis in children exposed to an adult sputum smear positive TB index case. As no randomized controlled study showing the benefit of this intervention has been performed, this decision is mostly based on the observation that this chemoprophylaxis is well-tolerated by the infants [11, 15, 20, 22, 23] but still lacking good evidence for prevention of TB disease.

The Swiss national guidelines recommend chemoprophylaxis with INH to all children 5 years or younger to prevent TB. In case of negative TST or IGRA 8 weeks after exposure, the prophylactic therapy can be stopped [7]. Similarly, the NICE Guidelines recommend INH prophylaxis but in case of negative TST and IGRA for 6 weeks after exposure only [3].

Given that at the time of diagnosis of the index case and start of the investigation, at least 6 weeks passed since the infant’s exposure of the NICU, we decided to waive the application of INH prophylaxis to all infants apart from her newborn. As a constant exposure until the diagnosis of the index patient was present in case of the index patient’s newborn and of the siblings, we followed the Swiss national guidelines as well as the NICE Guidelines and started isoniazid prophylaxis up to the second negative TST 2 months later.

Retrospectively, the repeated negative TST results of all hospitalized infants 6 months of age, where TST is considered to be reliable, support this approach. Likewise, the fact that the index patient’s newborn and her siblings were not infected and only one possible transmission among the exposed HCWs was observed, is reassuring.

### Secondary attack rate

Generally, transmission to newborns after exposure in medical facilities, seems to be rare: to date only three cases of infection [11, 18] and few cases of positive TST without signs of active disease have been reported [15, 17, 20, 21]. Crockett et al. found contaminated respiratory equipment to be the most likely source for the nosocomial transmission, whereas Steiner et al. found a nurse being the index patient having transmitted the disease to two infants [11, 18]. The secondary attack rate in this study including pediatric and adult contacts was 0.29% and 0.32% respectively in exposed versus tested individuals. No infant was diagnosed with TB infection. The here reported low secondary attack rate is consistent with earlier studies, where transmission is rarely described [2, 11–23].

There are different possible reasons for the low secondary attack rate observed in our investigation.

First, our index patient suffered from miliary TB with pulmonary involvement but not cavitary TB of her lungs. The literature regarding the contagiousness of patients suffering from miliary TB is scarce, but it is generally accepted that HIV TB co-infected patients presenting with a similar clinical picture, correspond to a paucibacillary infection and therefore are considered at low risk of transmission [25]. The fact that the mother as index patient did not even infect her newborn infant, as well as the two swiss-born and therefor non BCG-vaccinated siblings, where close and intense contact must be presumed, supports the hypothesis of low infectiousness of miliary TB.

Second, the mycobacterium identified in our index patient, was *M. africanum*. A recent study shows a reduced transmission rate for lineages of *M. africanum* compared to lineages belonging to *M. tuberculosis* [26]. Of note, *M. africanum* belongs to *M. tuberculosis* complex. Therefore, diagnostic tools as MTB complex PCR, TST and IGRA have similar diagnostic accuracy for *M. africanum* as for *M. tuberculosis* [27].

Third, the presumed risk of nosocomial transmission by HCWs is higher than by infants or relatives because of close and usually long-lasting contact between HCW and the exposed persons [2, 11–15, 20–22]. Our investigation, with only one potential transmission in the HCW cohort, and no transmission in the infant cohort, supports this hypothesis.

Fourth, the modern NICU environment with an active ventilation (6-7times/h) reduces the risk of airborne infections. Yet, Ahn et al. and Perry et al. report a low percentage of TST conversion in exposed infants in modern NICUs, showing persistent risk of transmission even in highly modern environments [15, 17]. This therefore justifies our decision regarding the contact definition of the infants.

One HCW showed a possible transmission. In this HCW the first IGRA was quantified with TB1/TB2 Antigen Nil 3.76/3.39 IU/mL. Interestingly a TST as a routine test 4 weeks before this IGRA was reported negative. This situation led to the speculation of a possible booster phenomenon. A booster phenomenon questions the reliability of a positive IGRA if following to a previous TST, due to the possibility of false positive IGRA result driven by tuberculin stimulation [28]. However, these phenomena are mostly seen in patients with previously positive TST, only few reports show a booster phenomenon in patients with a previous negative TST [29]. A second IGRA, conducted 19 weeks later showed a weaker stimulation (TB1/TB2 Antigen Nil 1.1/1.65 IU/mL), supporting the hypothesis of a booster phenomenon. In addition, as the TST was performed after constant exposure over 2 months, in case of transmission, a positive TST could have been expected at the time of investigation. Nevertheless, transmission could not be completely ruled out and diagnosis of LTBI was possible, therefore the HCW was treated for LTBI with rifampicin for 4 months.

### Viability of T-spot in contact infants

Current literature comparing the performance of IGRA (Quantiferon and T-Spot) and TST to identify TB infection in young children reveals limited evidence and conflicting results [5, 30–33]. Studies focusing on performance of T-Spot in comparison to TST may show discordant results with TST positive and T-Spot negative cases. However, the authors conclude that these conflicting results are based on overestimation of diagnosing LTBI using TST as a diagnostic tool. False positive TST cases, not seen in our cohort, are thought to result from recent BCG vaccination or non-tuberculous mycobacteria (NTM) exposure or infection. Yet, limited cell mediated immunity may lead to false negative T-Spot results, especially in children younger than 5 years. Given the absence of a gold standard for diagnosing LTBI in children, the validation of test performance is measured by progression to active TB disease although being a rare outcome. In this respect, a study published recently, evaluating the performance of IGRAs in children younger than 5 years, showed evaluable results in 98% of children and concluded that IGRA could be a useful tool to evaluate children at risk for TB [5]. The invalid rate was determined to be less than 1.8% for children younger than 12 months. In addition, a strong correlation between positive T-Spot results and well-recognized risk factors (e.g. burden of TB in the population) could be proven [5].

In line with this latest data, all infants in our outbreak investigation showed a negative TST and a concordant negative IGRA (T-Spot). All blood samples from infants, except one, responded to the positive control of IGRA (T-Spot), demonstrating that cells are viable and react upon stimulation by PHA. This implies the assumption that even premature infants may have reached a certain maturity of the immune system by the chronological age of 6 months, otherwise no positive control would be expected. These results may support previous literature, establishing T-Spot as a technically operational test in infants [5, 34]. Although our results should be eventually confirmed with samples from infants with TB infection, positive TST and a positive T-Spot.

### General remarks and challenges in TB diagnosis

The extent of our outbreak investigation shows the enormous consequences of a delayed TB diagnosis. Our case demonstrates the difficulty of TB diagnostics nowadays. Including diagnostic clues (such as patients country of origin), the accurate application of available tests and their correct interpretation is essential for a proper and prompt diagnosis of TB and, at the same time, represent a great challenge due to the investigator-dependence.

The development of simpler and faster point of care tests would certainly help to prevent such delays in diagnosis.

### Limitations

This investigation has several limitations. Given the limited data, contact definitions and management, as well as the protocol of our outbreak investigation were based on consensus opinions. We followed the NICE Guidelines as closely as possible (applicable).

We could not observe any proven transmission in our NICU cohort of infants, therefore the actual diagnostic accuracy of the T-Spot compared to TST cannot be verified.

## Conclusion

This investigation highlights the low transmission rate of sputum smear positive miliary TB, even in a highly susceptible population such as infants in a NICU. Our expert definitions and interventions proved to be useful in terms of feasibility of a thorough outbreak investigation. The repeatedly negative TST results retrospectively support the approach to waive INH-prophylaxis. Furthermore, we demonstrated concordance of negative T-Spot and TST results in infants. Based on these findings, we assume that T-Spot could be considered as a reliable investigation tool to rule out TB infection in infants. Further studies are needed to confirm the performance of T-Spot in TB infected infants.

## Data Availability

All authors had full access to the data.

## Abbreviations

NICU: Neonatal intensive care unit
TB: Tuberculosis
IGRA: Interferon gamma release assay
TST: Tuberculin skin test
PHA: Phytohemagglutinin
HCW: Health care workers
PCR: Polymerase chain reaction
MTB: *Mycobacterium tuberculosis*
INH: Isoniazid
LTBI: Latent tuberculosis infection
ELISA: Enzyme-linked immunosorbent assay
ELISpot: Enzyme-linked immune spot
